# Supplementary material for: Are carbon emissions trading and green financial instruments synergistic? -Comprehensive quantitative research based on content analysis
Source: PLoS One. 2024 Mar 7;19(3):e0298601. doi: 10.1371/journal.pone.0298601 (PMC10919601; doi:10.1371/journal.pone.0298601)
Supplement: S1 Data — (ZIP) [file pone.0298601.s001.zip › Data/地级市Z_F工作报告.zip!/╣π╬≈/║╪╓▌╩╨/└┤╫╘╣1⁄2╓┌║┼í╛┬φ┐╦╩2╛▌═°í┐_2004.docx]

各位代表：

　　现在，我代表市人民政府向大会报告工作，请予审议，并请市政协各位委员和其他列席人员提出意见。

2003年工作的回顾

　　2003年是我市撤地设市后着力打实基础、理顺关系、转变管理职能的开局之年，也是我市克服非典疫情、持续干旱以及电力供应紧张等不利因素影响，各项工作取得显著成绩的一年。一年来，新一届市人民政府以邓小平理论和“三个代表”重要思想为指导，深入贯彻落实党的十六大、十六届三中全会精神，坚持一手抓防治非典不放松，一手抓经济建设不动摇，以解放思想再讨论为动力，围绕财政增长、居民增收目标，以项目建设为载体，突出抓好招商引资，加快发展非公有制经济，着力推进工业化、城镇化、农业企业化，巩固提高商贸业和旅游业，经济发展明显加快，社会各项事业全面进步，完成或超额完成了市一届人大二次会议确定的各项目标任务。全市生产总值完成116.5亿元，比上年增长10.5%，实现了“九五”以来的最快增长；财政收入扭转了多年来徘徊不前的局面，完成7.06亿元，增长12.7%；全社会消费品零售总额35.7亿元，增长9.4%；全社会固定资产投资完成22.6亿元，增长83.4%，增幅居全区第一；外贸进出口总额1.17亿美元，增长29.6%；城镇居民可支配收入增长11.9%；农民人均纯收入增长5.4%；城镇登记失业率、人口自然增长率均控制在预期目标以内。

　　“三化两业”取得显著成效，经济结构进一步优化。大力推进“三化两业”，三次产业全面增长，一、二、三次产业分别增长5.1%、17.2%和10.6%，第二产业占GDP的比重由上年的27.8%提高到29.9%，成为经济增长的主要动力。坚持把发展工业摆在经济工作的首位，工业化取得新成绩。钟山和富川烟辅厂等工业项目建成投产，灵峰药业GMP认证等重点技改项目进展顺利，企业扭亏脱困成效显著，旺高工业区入园企业不断增多。全市规模以上工业企业实现增加值7.97亿元，增长21%；工业企业盈利1.38亿元，增长438.3%；规模以上工业企业经济效益综合指数155.5%，比上年提高55.7个百分点，创历史最好水平。大力实施城市建设“12345”工程，城镇化进程加快。全面开展城市总体规划和江南新区详细规划等规划的修编工作，完成南环路一级水泥路改建及路灯安装，改造维修八达西路，开工建设江北东路、平安大道等城市基础设施，落实“门前三包”责任制，开展“秋风”、“暴雨”市容市貌集中整治行动，城市品位和形象明显提升。加大“三农”工作力度，以推进农业企业化促进农业结构调整，培育发展了西牛牧业等36家新的农业企业，进一步带动了农业增产、农民增收，发展壮大了优势特色农业。扶贫开发取得新成效，贫困村屯的道路、人畜饮水等基础设施建设进一步加强，又有5320户、26694人解决了温饱问题。旅游业克服非典的不利影响，姑婆山、贺州石林、黄姚古镇、紫云洞等景点景区的开发建设步伐加快，旅游宣传促销力度加大，成功举办两广抗非典英雄聚贺州、贺州市（深圳）旅游推介会、全国旅游地理学会第18届年会等活动，全市接待国内外游客人数、旅游总收入分别增长18%和21%。商贸业得到巩固发展，一批专业市场建成使用或加紧筹建，商贸基础设施进一步改善，物流配送、连锁经营等现代流通业进一步发展。

　　百项工程大会战捷报频传，固定资产投资创历史最好水平。围绕推进“三化两业”，大力实施百项工程大会战，全市月月有项目开工、乡乡镇镇有项目上马。全年新开工项目448项，项目总投资35.6亿元，其中总投资3000万元以上的项目21个。桂东电子高压铝箔23条生产线等一批重大项目建成投产；桂梧高速公路贺州段、莲塘至鹰阳关二级公路、下福电站、城区电网建设与改造等重点项目开工建设；关系贺州发展全局的一批重大项目前期工作进展顺利，被视为贺州经济发展生命线的贺州至广州高速公路八步至灵峰段已获自治区批准立项。

　　各项改革取得新成效，招商引资实现新突破。国有企业改革继续深化，全市有119家中小企业实行了租赁、承包、兼并、破产、出售、股份制或公司制改革；平桂飞碟等一批国有企业通过改革重新焕发了生机；西湾矿务局关闭破产全面启动。在全区率先完成了农村税费改革任务。城镇职工基本养老保险制度和基本医疗保险制度进一步完善。农村义务教育管理体制、卫生管理体制、药品和粮食流通体制改革稳步推进。国有经营性土地使用权实行了招标拍卖挂牌出让，政府采购取得良好效果。整顿和规范市场经济秩序工作取得新成果。

　　深入开展“投资环境建设年”活动，规范行政事业单位收费秩序，减少行政审批事项404项，取消行政事业性收费项目83项，取消和降低行政事业性收费标准333项。设立市政务中心、外商投诉中心，开通市长热线电话，开展机关效能建设，制订损害软环境责任追究办法，改善了机关服务，提高了机关办事效率。部门招商、挂职招商、专业队招商、会务招商、以商招商等招商引资方式成效显著。全市共引进内资项目341个，利用外来资金15.7亿元，增长738%；引进外资项目27个，实际利用外资2061万美元，增长14.1%。进出口贸易在连续五年增长的基础上再创新高。

　　尽心竭力为群众办实事，“六大民心工程”深入民心。坚持执政为民，大力实施五保村建设、少数民族地区脱贫致富、大中专毕业生就业、农村富余劳动力培训就业、下岗失业人员再就业和社会稳定工程“六大民心工程”。全年新增就业岗位10592个，城镇登记失业率控制在自治区下达指标内。动工建设五保新村387个，建成232个，入住五保老人3145人，在全区率先走出了一条新时期农村五保老人集中供养的新路子。“两个确保”和“三条保障线”工作继续得到巩固和落实。突出加强治安力量，成立治安联防队，组织开展打击“两抢两盗”等专项集中行动，刑事案件下降，社会治安状况明显好转。金融保险业健康发展。城乡居民收入持续增长，消费水平提高，居住环境进一步改善。

社会各项事业全面发展，精神文明和民主法制建设得到加强。实施新一轮科技创新计划，科技与经济结合取得新成绩。“两基”教育成果得到巩固提高，中小学校布局调整和危房改造工作取得新进展，普通高中办学规模进一步扩大，梧州师专专科升本科、组建贺州高等职业技术学院工作稳步推进，各类学校办学条件进一步改善。人口与计划生育工作整体水平有新提高，人口出生率、自然增长率均控制在自治区下达指标内，计生工作在自治区的综合考评中，党政线考核荣获广西先进，四个县（区）计生工作再上新水平。抗击非典工作取得重大胜利，实现了无病例发生、无医务人员感染的目标。竞技体育取得良好成绩，全民健身运动广泛开展，成功举办了我市第一届运动会。文化艺术、广播电视、新闻出版事业健康发展，《贺州麒麟舞》等一批优秀文艺作品获得国家和自治区级奖项。以创建桂东文明绿洲为载体的精神文明创建活动深入开展。环境和资源保护进一步加强。人事、机构编制、工商管理、技术监督、药品监督、供销合作、民政、审计、统计、气象、人防、档案、修志以及安全生产、民族、宗教、外事、侨务、妇儿和残疾人等工作取得新成绩。依法治贺全面推进，各级政府和部门依法行政水平有新的提高。廉政建设取得新成效。国防教育、民兵和预备役建设以及双拥工作继续加强。全市社会稳定，民族团结，人民群众安居乐业。

　　各位代表，过去的一年，我们遇到的困难比预料的大，取得的成绩比预期的好。取得这样的成绩，是自治区党委、自治区人民政府和市委正确领导的结果，是各级人民代表大会及其常委会和人民政协监督支持的结果，是全市人民团结拼搏、开拓进取的结果。在此，我代表市人民政府，向全市各族人民，向驻贺人民解放军和武警部队官兵，向各民主党派、工商联、人民团体以及所有关心支持我市各项事业建设的各界人士，表示衷心的感谢并致以崇高的敬意!

回顾过去一年的工作，我们有五点体会尤为深刻：

　　一是全市上下团结拼搏，紧紧抓住经济建设这个中心，一心一意谋发展，凝神聚力抓建设，是我们取得所有成绩的根本所在。尽管遇到了比预想中大得多的困难，但全市上下始终坚持把加快发展作为第一要务，团结拼搏，迎难而上，推进了经济快速发展和社会各项事业全面进步。

　　二是解放思想，开拓创新，及时果断调整阶段性经济工作重点和目标，是实现经济快速增长的关键。在深入调研和广泛听取群众意见的基础上，我们提出以财政增长、居民增收为基本目标，以项目建设为载体，以招商引资和发展非公有制经济为突破口，着力推进“三化两业”，实现经济持续快速健康发展的阶段性工作重点和财政工作三年根本好转、城市建设五年创新城的目标，并制定和实施百项工程大会战等一系列具体措施，使得经济建设的各个领域打开了新局面，实现了新突破。

　　三是坚持以项目为载体，努力扩大对外开放，实现招商引资新突破，是促进经济快速增长的重要途径。我们以解放思想再讨论为动力，把招商引资、引进项目、扩大投资规模作为促进经济增长的重大举措，创新招商方式，以粤港澳地区为重点，开展部门招商、专业队招商、挂职招商等多形式招商引资，引来了大批客商、资金和项目，借助外力有效地促进了经济增长。

　　四是切实转变作风，强化服务，及时解决工作中存在的问题，改善投资环境，是加快经济发展的前提。我们狠抓工作落实，定期对经济工作督促检查，及时协调解决发展中的问题，强化政府的服务职能，开展机关效能建设，减少行政审批事项，提高行政效率和服务水平，改善了投资环境，为经济快速增长和社会全面进步创造了良好条件。

　　五是强化激励机制，落实经济目标责任制，是经济快速增长的有力保障。我们制订了经济目标责任制、重大项目责任制，出台了鼓励企业投资优惠政策和激励招商引资、出口创汇等办法，把工作落实到各级各部门、每个机关干部，充分调动方方面面的积极性，形成了加快发展的合力。

　　在看到成绩的同时，我们也清醒地看到，当前发展中仍然面临不少困难和问题，主要是：经济总量偏小、结构不合理、运行质量不高，工业的支撑作用还不够强；财政困难的局面仍未根本缓解；农业结构调整和农业增效的任务较重，农民增收门路少，就业压力大，部分群众生活困难；发展的软硬环境还有待进一步改善，特别是交通、服务和社会治安环境建设还要进一步加强；撤地设市后，许多重大关系尚未完全理顺，政府管理职能、干部的工作方式方法转变尚未完全到位。对这些困难和问题，我们将在今年的工作中，采取积极措施，努力加以解决。

2004年政府工作的总体要求和主要任务

　　今年是我市加快推进“三化两业”进程，实现五年阶段性工作目标的关键一年，政府工作的总体要求和主要任务是：以邓小平理论和“三个代表”重要思想为指导，全面贯彻落实党的十六大和十六届三中全会精神，抓住中国──东盟博览会落户南宁、泛珠江三角洲经济区域合作加强和西部大开发等重要发展机遇，抓好发展这个第一要务，坚持科学发展观，按照“五个统筹”的要求，继续以财政增长、居民增收为基本目标，以项目建设为载体，以发展非公有制经济和外向型经济为突破口，以加快推进工业化、城镇化、农业企业化，巩固提高商贸旅游业为重点，以打通道、降门槛、引项目、建基地为手段，进一步深化改革，扩大开放，切实抓好就业和再就业，着力解决事关人民群众切身利益的突出问题，大力发展社会各项事业，加强精神文明和民主法制建设，正确处理改革发展稳定的关系，推进经济社会全面、协调、可持续发展，实现社会主义物质文明、政治文明和精神文明共同进步。

　　今年全市经济社会发展的主要预期目标是：全市生产总值增长10%，财政收入增长12%，全社会固定资产投资增长35%，全社会消费品零售总额增长12%，外贸出口增长10%，实际利用外资增长10%，农民人均纯收入增长5%，城镇新增就业6000人，城镇登记失业率控制在4.5%以内，人口自然增长率控制在7.85‰以内。确定上述预期目标，体现了积极稳妥和加快发展的要求，是积极可行的，经过努力是完全可以实现的。在实际工作中，还要按更高的目标要求去努力。要实现这些目标，必须突出加快发展主题，以发展解决前进中的问题；必须把握经济结构调整的主线，加快推进“三化两业”，注重提高经济增长的质量和效益；必须深化改革，扩大开放，以开放促改革、促发展；必须牢固树立和落实科学的发展观，按照“五个统筹”的要求，推进经济持续快速健康协调发展和社会全面进步；必须更加注重以人为本，以维护最广大人民群众的根本利益为工作的出发点和落脚点，不断提高人民群众生活水平；必须加强政府自身建设，加快政府职能转变，狠抓工作落实，着力提高政府管理经济工作的水平。

今年要重点做好十个方面的工作：

　　一、加快推进工业化，培育和壮大经济支柱

坚持把发展工业摆在经济工作的首要位置，按照“大改革、大企业、大群体、大产业”的思路，编制并落实好工业发展总体规划，做大做强传统优势产业，培育发展新的优势产业，使工业尽快成为振兴经济、振兴财政的最强有力的支撑。

　　加大技术改造力度，发展壮大现有优势产业。围绕发展壮大电力、林产、电子、制药、建材、矿产、食品等优势产业, 实施一批工业重大技改项目，力争技改投入增长30%以上。年内要完成桂东电子中高压铝箔新增37条生产线的建设，继续抓好灵峰药业GMP认证和年产50万箱金鸡系列中成药技改项目二期工程；加快把桂东电子、梧州松脂等企业培育成年销售收入5亿元以上、创利税3000万元的企业，把贺州电业公司、贺达纸业、灵峰药业培育成年销售收入10亿元以上、创利税亿元以上的企业。各县（区）也要加快培育形成3—5家收入超亿元、创利税超千万元的企业。

　　开工建设一批新项目，培育新的优势产业。重点整合林业、矿产和农业资源，全力推进林浆纸一体化、石材工业和农副产品加工业等重大项目的前期工作和组织实施工作。年内争取开工建设贺达纸业10万吨高级文化纸、上程电站、大锰银鹤电池有限公司年产1.2亿只电池等项目。加紧筹建石材加工销售基地，争取年内一期工程基本建成。启动建设2万吨果汁饮品、年加工500吨脐橙打腊电光分级保鲜、年产300吨高档成品茶、1万吨精炼茶油等农产品加工项目。继续引进、发展壮大服装、玩具等劳动密集型产业。

　　加快工业园区建设，建好产业发展载体。启动全市工业发展总体规划编制工作，拓宽筹资渠道，多元投入，加快旺高工业园区基础设施建设，改善园区创业条件，增强吸纳项目的能力。坚持以招商引资为重点，创新招商形式，吸引更多的外来资本、民间资本特别是东部转移产业进入园区，努力把工业园区建成接纳东部产业转移的重要基地，成为工业经济新的增长点。

　　以产权制度改革为核心，全面深化国有企业改革。以促进国有资产保值增值、妥善安置职工为目标，认真贯彻落实国家和自治区有关国有企业改革的配套政策和措施，加大国有企业改革力度，实行一企一策，分类指导，成熟一个，改制一个，通过整体出售、破产收购、兼并重组和民营化等各种有效形式，放开搞活现有中小企业。今年年底前全市基本完成国有中小企业改制工作。重点抓好梧州松脂上市辅导期内的规范调整完善工作，争取尽快上市。深化企业国有资产管理体制改革，整合国有资产，提高运行效率。加强服务和协调工作，努力为企业的生产经营创造良好的条件。

　　二、加快推进城镇化，扩大城市规模，提高城市品位

　　大力实施城市建设“12345”工程，按照高起点规划、高标准建设、高效能管理、高水平经营的要求加快城市建设，提高城镇化水平。

　　进一步完善城市规划，按照规划建设和管理城市。今年要完成贺州市城市总体规划修编、江南新区以及其他小区的详细规划编制工作，同时，着手开展城市风貌规划，抓好姑婆山旅游城等规划的制订完善，为今后城市建设发展提供科学依据。继续实行公众参与规划编制，增加规划编制的科学性和透明度。加强规划实施的管理，依法确保规划全面、有效实施，严格按照城市规划进行城市建设。

狠抓城市路网建设，大力改善交通条件。把今年作为“交通建设年”，加强道路建设，大力改善市内交通主干道和县乡、乡村的路网结构和路况，提高与周边地区的通达能力，解决好交通瓶颈问题。继续协助做好洛湛铁路贺州段开工的准备工作。加快桂梧高速公路贺州段以及鹰阳关至莲塘、富阳至麦岭二级公路建设。争取开工建设广贺高速公路八步至灵峰段、昭平县城至蒙山黄村二级公路和富川柳家至钟山、恭城三级公路。抓好一批县乡公路的建设。

　　加大城市配套设施建设力度，打造城市新面貌。认真抓好城市建设“两年一小变”方案的组织实施。年内要完成贺州大道、建设路的改造，搞好绿化、美化、亮化和净化，把这两条道路打造成代表贺州新形象的城市大道。开工建设江北西路，拓宽改建平安东路等道路，改善城区路网及路况。以桂梧高速公路延长线建设为契机，启动江南新区建设。加快灵峰花园等住宅小区和房地产项目建设，建设一批经济适用住房。启动西约街片的旧城改造。开工建设市防洪排涝一期工程、垃圾处理厂以及地下管网工程，建设市客运中心，筹建新博物馆和文化体育中心，继续抓好龟石供水工程等重点工程续建，完善城市基础设施。加快发展城市公共交通，通过引进外资和鼓励民间资本投入，抓好一批公交场站、候车亭建设，增加公交运营车辆和线路，提高公交运营车辆档次，逐步建立起与城市发展水平相适应的公共交通体系。加快各县城路网、供电、供水等基础设施以及住宅区建设，抓好信都、望高、黄姚、朝东等重点镇的建设。鼓励农民进城投资置业，大力推进城镇化。

　　充分运用市场机制，提高经营城市水平。坚持政府主导、市场运作，推进国有土地的资本运营。要建立和完善土地储备制度，对经营性用地全部实行招标拍卖、挂牌出让，提高城市土地的使用效益。发挥城市建设投资公司在城市开发建设和经营中的作用，积极争取国债、银行贷款和社会资金等各类资本参与城市建设、经营和管理。大力推进城市基础设施等有形、无形资产的资本化运作，通过出让、拍卖、租赁经营权、冠名权、广告权和管理权，尽可能多地通过市场解决城市建设和管理资金，实现城市资源效益最大化。

　　加强城市管理，规范城市秩序。尽快建立和完善城市管理综合执法机制，加大执法力度，大力开展城市容貌、环境卫生、交通秩序、市场经营等专项整治行动，加强市容市貌管理，努力创造洁净、优美的城市生活环境。

　　三、加快推进农业企业化，重视和解决“三农”问题，努力增加农民收入

　　把解决“三农”问题作为我们全部工作的重中之重，按照“多予、少取、放活”和统筹城乡发展的要求，采取更直接、更有力的措施，加强农业、支持农业、保护农业，加快推进农业企业化，大力调整农业结构，发展壮大县域经济，努力增加农民收入。

　　大力培育发展农业企业，做大做强优势特色农业。要运用经营工商企业的理念来发展农业，把推进农业企业化作为解决“三农”问题的重要手段和发展农村经济的载体，以企业带基地，以基地连农户，实现集约化经营。要落实扶持农业龙头企业的政策措施，支持梧州松脂、贺达纸业等重点农业龙头企业进一步做大做强。加大招商引资力度，引进、发展一批新的农产品加工、流通龙头企业。在保证粮食安全的前提下，引导农民按市场需求调整种养业结构，大力发展优质、高产、高效、生态和无公害农产品，突出抓好脐橙、沙田柚、梅李、烟叶、茶叶、马蹄、红瓜子、蚕桑、无公害蔬菜等特色农产品基地建设，着力打造有贺州特色的优势农产品品牌，提高农产品质量和竞争力。今年要启动20万亩脐橙生产基地的建设，加快发展脐橙产业，努力把我市建设成为中国著名的脐橙产区。积极发展畜牧水产业，进一步发展瘦肉型猪、优质肉牛、奶水牛、家禽以及昭平桂江名优鱼、钟山黄沙鳖繁育等基地，提高畜牧水产业在农业中的比重。加强林业生态工程建设，加快建设速生丰产林、用材林、松脂林基地，加强生态公益林的建设与保护。全面推进无公害农产品行动计划，抓好无公害农产品标准化生产，做好八步无公害蔬菜、富川无公害水果、昭平无公害茶叶和钟山无公害优质稻生产示范基地县(区)的申报认定工作。积极发展各类农村专业合作经济组织，发挥它们在农产品销售中的积极作用，确保农产品销售渠道的畅通。

　　统筹城乡发展，发展壮大县域经济。要把加快发展县域经济作为解决“三农”问题、增强各县（区）经济实力、缓解县乡财政困难的重要措施，抓住发展非公有制经济和特色经济这两个关键，推进农村工业化、农业企业化和农村城镇化。放手发展非公有制经济，通过各种措施激活民间资本，使非公有制经济尽快成为县域经济发展的主体。大力发展农村工业，与推进农业企业化相结合，调整改造提高乡镇企业，大力发展农副产品加工业，扶持一批骨干企业，培植优势主导产业和特色产业，提高工业在县域经济中的比重，以工业化带动城镇化。加快推进农村城镇化，通过政策引导，运用市场机制鼓励社会资金投入小城镇开发，积极发展小城镇经济，发展农村二、三产业，使小城镇成为县域经济发展的增长点。乡镇经济是县域经济的基础，要理顺权责利的关系，充分调动乡镇的积极性，加快发展乡镇经济。

　　加强农村基础设施和社会化服务体系建设，改善农村生产生活条件。要努力增加对“三农”的投入，继续加强农田水利基本设施和农村市场、道路、供水、供电等基础设施建设，大力实施好龟石水库灌区配套与节水改造、农村人畜饮水、小水电代燃料、农村水电电气化、农网改造和农村生态能源等工程，进一步改善农民的生产生活条件。加快农村科技推广体系建设，完善县乡农技推广网络；加快建立市、县（区）、乡（镇）三级农产品质量安全监测体系，继续加强市农产品质量安全检测分中心以及市动物产品、饲料兽药检测中心建设，开展农产品检测工作，开辟农产品销售的“绿色通道”。做好动、植物疫病防治工作。要继续推进农村税费改革，认真落实国家取消除烟叶外的农业特产税以及从今年起逐步降低农业税税率、5年内取消农业税的政策措施，切实减轻农民负担。深化粮食流通体制改革，全面放开粮食购销市场，认真落实国家对农民实行粮食补贴的政策措施。加强农业生产资料价格监管，保护农民利益。要实行最严格的耕地保护制度，依法加强耕地管理，坚决制止乱占滥用耕地，纠正随意改变基本农田用途的现象。完善土地征用、征收程序和补偿机制，积极推进土地承包经营权合理流转。鼓励农民外出务工经商，搞好劳务输出，取消对农民进城的不合理限制，改善农民进城就业环境，努力增加农民的非农收入。继续实施农村富余劳动力就业培训工程，增强农民的就业和创业能力，多渠道扩大农村劳动力转移就业。要改进农村金融服务，农村信用社要增加农户小额信用贷款和农户联保贷款，支持农民发展生产。继续抓好少数民族地区脱贫致富工程，搞好扶贫开发，多渠道增加扶贫资金投入，扩大以工代赈规模，使农村未脱贫人口尽快解决温饱问题。

　　四、巩固提高商贸旅游业，加快发展第三产业

　　以商贸旅游业为重点，加快发展服务业，进一步提高服务业在国民经济中的比重。

　　围绕建设桂东历史文化名胜自然生态旅游区，做大做强旅游业。按照自治区提出的建设桂东历史文化名胜自然生态旅游精品品牌的要求，完成全市旅游总体规划编制工作，进一步做好姑婆山旅游城、富川秀水状元村景区和钟山荷塘十里画廊风景区的规划，采取政府推动、市场引导、企业运作的方式，引进有实力的旅游企业参与旅游资源开发。着力搞好姑婆山、大桂山、贺州温泉、贺州石林、黄姚古镇、紫云洞、碧水岩、秀水状元村等景点景区的开发建设，提高旅游景点景区档次。加强旅游配套设施建设，完善行、住、吃、游、购、娱等设施，抓好旅游产品开发，进一步延长产业链。全面推行旅游行业接待服务标准化管理，继续抓好旅游从业人员的培训，提高旅游业接待服务水平。加大宣传促销力度，加强与周边地区旅游市场的协作，主动融入粤港澳和桂林旅游圈，扩大客源市场，提高贺州旅游的知名度和吸引力。

　　大力加强市场建设，加快发展商贸业。以有形市场建设为重点，推动商贸业的发展，努力使我市成为桂东地区重要的商品集散地和物流中心。加快建设各类专业批发市场、连锁超市等商贸设施，重点抓好湘粤桂农贸市场、建材市场、城西市场、小五金市场、小商品市场等一批专业市场的建设。改善商贸基础设施。积极引进大型贸易流通企业，不断提高流通企业的规模化、组织化和规范化水平。

积极发展新兴服务业，完善服务业体系。深化住房制度改革，全面放开住房二级市场，搞好经济适用房建设，发展规范房地产中介服务，重视房地产管理，积极培育房地产交易市场。鼓励民间资本参与现代服务业的发展，积极发展教育培训、文化娱乐、卫生保健、体育健身、社区服务等新兴服务业，进一步发展信息、会计、监理、咨询、法律服务、科技服务等中介服务业。策划实施“数字化贺州”工程，搞好信息基础设施和政府、企业与公共领域信息网络建设，合理开发利用信息资源。

五、深入实施百项工程大会战，保持投资持续快速增长

　　强化“抓项目就是抓发展”的意识，按照“续建一批、新开工一批、竣工一批、储备一批”和“大、中、小项目一起上”的要求，深入实施百项工程大会战，积极推进项目建设，努力扩大投资规模，保持投资持续快速增长。

　　推进一批重大项目，扩大投资规模。今年列入全市百项工程计划的新开工重点项目141个，总投资超过152亿元；续建项目71个，年度计划投资21亿元。对计划开工项目，要加大组织协调力度，力争按计划开工建设，确保月月有项目开工、季季有项目竣工。对在建项目，切实加强项目管理，加快建设进度，按投资计划完成投资。

　　加快项目前期工作，精心储备好项目。围绕推进“三化两业”，加大项目前期工作投入。根据我市资源优势，精心研究、推出和储备一批能够提高资源增值率的资源型加工项目、带动全市经济结构优化升级的重大工业项目、改善我市投资环境的基础设施项目，抓住国家投资向农村、结构调整、中西部、生态环境建设倾斜的机遇，抓紧申报，争取更多地挤进国家和自治区投资的计划盘子。认真研究国内外投资形势，有针对性地筛选推出一批招商引资项目，争取更多的国内外投资。

　　拓宽资金筹措渠道，努力开创多元投资新格局。深化投融资体制改革，创新投资和项目建设机制，加快引进和培育壮大多元投资主体，大力拓宽民间投资渠道和企业融资渠道，积极引导企业面向资本市场直接融资，面向国际国内市场寻求合作伙伴，更多地引进国外市外资金。积极争取国债资金、中央预算内专项资金、政策性贷款等国家各类资金支持。加快推进基础设施投资经营市场化，形成基础设施建设的良性循环机制。放宽市场准入，打破部门、行业投资经营垄断，吸引市内市外、境外和民间资本投资基础设施建设。加强投资和项目建设管理，坚持以制度保证资金安全使用，确保工程质量。

　　六、进一步扩大对外开放，大力发展开放型经济和非公有制经济

　　以中国─东盟博览会落户南宁、泛珠江三角洲经济区域加强合作、东部产业转移和西部大开发为契机，深入实施开放带动战略，加大招商引资和外贸出口力度，积极稳妥地推进各项改革，加快发展开放型经济和非公有制经济，促进我市经济快速发展。

　　努力实现招商引资新突破。继续实行招商引资目标责任制，在抓好部门招商、专业队招商、挂职招商等传统招商形式的基础上，进一步探索行之有效的招商引资新措施、新办法。积极参与泛珠三角区域经济合作，特别要加强与广东佛山市的经贸合作，抓好旺高工业区佛山工业城的建设。进一步加强与广东美的集团合作，引进和投资开发一批产业项目。做好贺州市桂东电子产品订货会暨经贸项目洽谈会已签约项目的跟踪落实工作，确保项目如期开工建设和资金落实到位。

　　千方百计扩大外贸出口。实施科技兴贸战略，进一步调整优化出口产品结构，重点提高松脂、金属硅、锡锭等资源型加工出口产品的技术含量及其附加值，增强出口商品竞争力。积极发展出口导向型企业，推动进出口经营权向中小企业、个体私营企业扩散，壮大出口主体队伍。抓好出口协调服务，在加强银贸、税贸、关贸等协调服务的同时，逐步建立和完善贸易促进会、同业协会等符合国际惯例的贸易促进体系。

　　加快发展非公有制经济。把发展非公有制经济摆到更加突出的位置，扫除非公有制经济发展的体制性障碍，形成政府鼓励创业、群众自主创业的良好氛围。放宽市场准入，允许非公有资本进入法律法规未禁入的基础设施、公用事业及其他行业，使非公有制企业拥有与其他企业同等的投资机会。改革审批限制，消除政策歧视，使非公有制企业在注册、经营等方面得到与其他企业一样的行政服务和市场环境。依法保护私有财产，使非公有制企业实现与其他企业平等的权益保障。通过营造更为宽松的政策环境和公平竞争的市场环境，促进非公有制经济更快更好地发展。

　　七、培植壮大财源，增强财政实力

　　围绕“一年缓解、两年改善，三年根本好转”的财政工作目标，加大财源培植力度，深化财政体制改革，增强财政实力，优化支出结构，强化财政职能。

　　大力培植和开拓财源，加强税收征管，扩大财政总量。结合经济结构调整，通过培植利税大户、搞好国有资产经营、盘活闲置资产、做好经营城市和以地生财文章、发展创税农业、加强预算外资金调节与监管等措施，完善激励机制，调动各方参与财源建设的积极性，拓宽增收渠道，增加财政收入。努力降低非税收入比重，把财政增收建立在税收增加的基础上，优化财政收入结构。进一步理顺县乡财政体制，加大财政转移支付力度，逐步改变困难乡镇的财政状况。坚持依法治税，加大征收稽查监督力度，确保税收应收尽收，努力把经济发展成果反映到财政收入上来。

　　优化支出结构，努力实现财政收支平衡。继续按照保工资、保稳定、保法定支出的顺序安排预算，进一步调整支出范围，合理确定支出定额，严格控制一般性支出，压缩一切非必要的和不合理的开支，集中财力保重点、办大事，提高财政支出效益。继续深化部门预算改革，推进国库集中收付制度改革。完善政府采购制度。严格工资专户管理，财政资金要优先保证发放工资。进一步强化财政管理和监督，整顿和规范财经秩序。

　　八、抓好就业和社会保障工作，提高城乡居民生活水平

把事关人民群众切身利益的就业和社会保障工作摆上更加突出的位置，继续实施“六大民心工程”，千方百计扩大就业，进一步完善社会保障体系，着力解决群众生产生活中的突出问题，让人民群众生活水平在改革发展中不断得到提高。

　　大力促进就业和再就业。坚持劳动者自主择业、市场调节就业和政府促进就业的方针，把改善创业环境、增加就业岗位作为政府的重要职责，立足于发展经济扩大就业。落实好国家有关税费减免、社保补贴、小额担保贷款等扶持就业和再就业的各项政策，扎实推进大中专毕业生就业、下岗职工再就业工程，加快劳动力市场建设，完善就业服务体系，做好劳务输出工作。鼓励扶持发展以非公有制经济为主体的劳动密集型产业、服务业、中小企业，扩大就业门路，增加就业岗位。引导人们转变就业观念，以多种形式和灵活方式自谋职业、自主创业。

　　建立健全社会保障体系。继续做好“两个确保”和城市“低保”工作，健全城市居民最低生活保障制度，落实好城市低保标准和保障对象范围政策。实行重点监控和调剂制度，确保国有企业下岗职工基本生活费、参保企业离退休人员基本养老金按时足额发放，防止发生新的拖欠。依法扩大社会保险的覆盖面，重点抓好个体私营等非公有制企业和灵活就业人员的参保工作，依法把应保而未参保的企业和职工逐步纳入社会保险。加强对各类参保企业社会保险费的征缴，提高征缴率。加强社会保障资金的管理。完善和规范企业退休人员社会化管理工作。抓好医疗保险扩面工作，推动工伤保险和生育保险的发展，力争年内全市城镇职工医疗保险参保人数达到7.3万人。

　　切实解决群众关心的难点问题。完善社会救助和法律援助制度，做好清理拖欠农民工工资工作，维护弱势群体的合法权益，切实帮助城乡特殊困难群众解决就医看病、子女上学、无力打官司等实际困难。继续实施好农村五保村建设工程，确保年内完成80%以上的五保村建设任务。要抓紧解决城镇房屋拆迁和农村土地征用中存在的问题，在农村征用土地中，严格依法按规划和程序征地，及时给予农民合理补偿，切实保护农民合法权益。

　　九、大力发展各项社会事业，促进经济与社会协调发展

树立和落实科学的发展观，统筹推进各项社会事业全面进步，实现经济社会全面、协调、可持续发展。

　　大力推进科技进步。加大新一轮科技创新计划的实施力度，不断促进科技与经济的紧密结合。深化科技体制改革，继续加强农村科技工作，逐步建立和完善工业科技、城市科技体系。围绕电力、林产、制药、食品等优势产业，加快新技术、新品种的引进、研制、开发，应用高新技术改造和提升传统产业。扶持和发展一批科技中介服务机构，开展社会化、多样化、专业化服务。加强市县（区）现代农业科技示范园区及畜牧、水产等各类科技示范、转化、产业化基地的建设。广泛深入开展科普活动，提高全社会科技意识。

　　加快发展教育事业。实施科教兴市和人才强市战略，切实把教育放在优先发展的地位，投入更多的精力、更多的财力加快教育事业发展。坚持以全面推进素质教育、全面提高教育教学质量为中心，大力实施“塑造教育形象工程”、“统筹城乡教育发展工程”和“职教中心建设工程”，突出抓好师德师风教育、中小学校危房改造和安全稳定工作，扩大高中阶段教育规模，加快信息技术建设步伐，深入治理教育乱收费，积极推进教育教学改革，全面推进素质教育，不断提高教育教学质量。加大投入力度，积极创造条件做好梧州师专专科升本科工作，确保贺州学院申办成功。加快筹建贺州高等职业技术学院。积极鼓励社会力量办学，规范和发展民办教育，逐步完善多元办学体系。树立科学的人才观，加强人才工作，大力培养和引进高层次人才和我市急需的各类紧缺人才。加快人才政策法规建设，健全各类人才的选拔任用、考核评价、激励监督机制。大力优化人才服务环境，加快发展和完善人才市场，促进人才合理流动。努力营造鼓励人才干事业、支持人才干成事业、帮助人才干好事业的社会环境。

　　大力发展卫生事业。加强公共卫生体系建设，全面做好计划免疫和各种疾病的防治工作，提高应对重大传染病等突发公共卫生事件的能力。推进广济医院股份制改革，抓好市县疾控中心和传染病医院（科）、市紧急救援中心等工程建设，完善医疗设施。加强农村医疗卫生基础设施和农村卫生队伍建设，完善县、乡、村三级医疗卫生服务网，改善农村医疗卫生条件。做好新型农村合作医疗制度试点工作。实施好母亲安全工程。继续抓好直接关系人民群众身体健康和生命安全的食品、药品等方面的专项整治。同时，在城乡广泛开展爱国卫生运动。

　　发展文化等各项事业。实施文化精品战略，打造反映时代精神和具有地方特色的文化品牌。筹办好贺州市麒麟艺术节。做好中国海陆丝绸之路贺州古道文化资源的挖掘、整理和开发工作。积极发展文化产业，实现经济与文化发展的良性互动。继续加强基层文化建设，巩固和建设好乡镇图书馆和乡镇、村宣传文化站（室），丰富城乡群众文化生活。整顿和规范文化市场秩序，加强网吧、音像市场、娱乐场所和演出市场的管理。加强新闻出版工作，深入开展扫黄打非，净化出版物市场和文化市场。实施自然村通广播电视工程，加强广播电视基础设施建设，扩大广播电视的有效覆盖。广泛开展全民健身运动，加强体育人才的培养，提高竞技体育水平，发展体育产业。发展气象、社会福利等事业。认真做好人民防空、防震减灾工作。关心和支持老龄、妇女、儿童、残疾人等社会事业。继续重视做好对台、外事、侨务、民族、统计、档案等工作，推进各项事业发展。

统筹人与自然和谐发展，增强可持续发展能力。全面贯彻落实国家人口与计划生育政策法规，加强基层基础网络建设，广泛开展优质服务活动，加大对出生人口性别比偏高问题的专项整治力度，高度重视对流动人口的管理和服务工作，逐步建立和完善计划生育利益导向和社会保障机制，进一步稳定低生育水平。依法加强资源管理，合理开发利用资源。加快建立和完善土地储备制度，加大处置闲置土地力度，促进土地的合理、高效和可持续利用。加强生态建设和环境保护，推进以农村沼气为重点的生态能源建设，抓好水土保持综合治理。强化环境保护目标责任制的落实，切实加强城市环境综合整治，重点整治机动车辆、金属加工、建筑施工、修理业、娱乐业的噪声污染以及饮食服务业的烟尘污染等问题。加大城市生活污水的整治和工业污染源的防治力度，抓好贺江“清新行动”的实施工作，整治贺江污染，改善贺江水环境质量。加强环境执法，严肃查处环境违法行为。

　　十、加强社会主义精神文明和民主法制建设，为经济和社会发展营造良好环境

　　大力加强精神文明和民主法制建设，努力为经济社会发展提供精神动力和法制保障，推动物质文明、政治文明和精神文明协调发展。

　　积极推进精神文明建设。继续深入开展“三个代表”重要思想的学习教育活动，切实用邓小平理论和“三个代表”重要思想武装头脑、指导实践、推动工作。以创建桂东文明绿洲为载体，以提高市民素质和提升城市形象为目标，扎实开展文明村镇、文明行业、文明单位等群众性精神文明创建活动，引导城乡居民形成科学、健康、文明的生活方式。加强信用体系建设，抓紧建立企业和个人信用信息征集体系、信用市场监督管理体系和失信惩戒制度，努力打造“诚信贺州”，创造良好的信用环境，为贺州经济发展服务。加强科普宣传教育，大力弘扬科学精神。加强爱国主义教育基地建设，为开展精神文明活动提供有效载体。做好“双拥”共建和优抚安置工作，搞好全民国防教育，加强国防动员、民兵和预备役工作。

　　进一步加强民主法制建设。健全民主制度，保障人民群众的民主权利。认真执行市人大及其常委会的各项决议、决定，自觉报告政府工作和接受监督。积极支持人民政协履行政治协商、民主监督和参政议政职能，认真听取民主党派、工商联及各人民团体的建议和意见。同时，接受新闻舆论监督和社会公众监督；认真办好人大代表议案、建议和政协委员提案。巩固和扩大基层民主，不断完善村民自治管理、社区民主管理和企业民主管理制度，进一步推进政务公开、村务公开、厂务公开。深入开展各种形式的普法教育，在全社会营造学法、懂法、守法的良好环境。

　　切实抓好社会稳定工作。继续实施社会稳定工程，进一步落实社会治安综合治理各项措施，推进社会治安防控体系建设。严厉打击“两抢两盗”、黑恶势力、严重暴力犯罪、多发性侵财犯罪，坚决扫除“黄、赌、毒”等社会丑恶现象，严密防范和严厉打击“法轮功”邪教组织破坏活动和各类恐怖活动，切实维护社会稳定，保障人民群众安居乐业。严厉打击制售假冒伪劣产品、非法传销和商业欺诈行为。正确处理新形势下的人民内部矛盾，重视做好信访工作，认真抓好矛盾纠纷尤其是土地、水利和山林纠纷的排查调处，疏导化解矛盾，积极预防和妥善处理群体性事件。加强国家安全保密工作。完善和落实安全生产各项政策措施，继续开展以矿山、危险化学品、易燃易爆品、公共聚集场所消防、道路交通和农机安全生产为重点的安全生产专项整治，完善安全生产设施，认真排查各种事故隐患，强化安全生产管理和安全监察，严格执行安全生产责任制，避免重特大安全事故发生。

进一步加强政府自身建设

　　适应新的形势、新的任务，按照“廉洁、勤政、诚信、务实、高效”的要求，切实改进政府工作，推进行政管理体制创新，提高行政效率和服务水平，开创政府工作新局面。

　　转变职能，建设服务型政府。按照与国际惯例接轨、适应社会主义市场经济体制的要求，加快行政行为和管理方式改革，切实把工作的着力点放到经济调节、市场监管、社会管理和公共服务上来，进一步把不该由政府管的事交给企业、社会组织和中介机构，更大程度地发挥市场在资源配置中的基础性作用。根据自治区的统一部署，推进政府机构改革和事业单位特别是乡镇事业单位改革，切实提高行政效能。大力推进行政机关效能建设，建立健全行政效能评估机制，提高政府部门及其工作人员服务效率和质量。加强政府信用建设，认真兑现和落实承诺的事项，增加政府的诚信度，着力打造“诚信政府”。完善政务中心服务功能，全面推行一次性告知制、首问负责制、服务承诺制、限时办结制，推行集中审批和联合审批，精简审批事项，简化办事程序，提高服务质量和办事效率。加快建立健全各种突发事件应急机制，提高政府应对各种公共危机的能力。

　　依法行政，建设法治化政府。各级政府要依照依法治市的要求，推进行政行为法治化，按照法定权限和程序行使权力、履行职责。认真做好行政许可法的学习、宣传和贯彻落实工作，按照要求完成行政许可事项、规定和实施主体的清理工作。建立健全行政执法责任制、行政评议考核制、行政执法错案责任追究制、行政赔偿制和执法公示制，促进严格执法、公正执法和文明执法，做到有法必依、执法必严、违法必究。建立健全政务公开制度、重大问题集体决策制度、专家咨询制度、社会公示和听证制度，对涉及国民经济和社会发展的重大问题广泛征求民意，增强政府决策的透明度和公众参与度，进一步提高科学民主决策水平。建立健全决策责任制度，对不依照法定权限、违反法定程序、损害国家和人民利益的决策行为，要严肃追究责任人的法律责任。

　　与时俱进，建设学习型政府。建立和健全公务员和领导干部学习制度，加强理论学习和业务培训，不断加强理论武装、知识更新和能力培养。大力弘扬理论联系实际的学风，用心做事，用智慧办事，把理论用于实践，在实践中掌握新知识、积累新经验、增长新本领，不断提高科学判断形势的能力、驾驭市场经济的能力、应对复杂局面的能力。要加强公务员队伍建设，提高公务员素质，深入开展公务员行为规范教育和“人民满意公务员”评比、推荐、表彰活动，推行公开选拔、竞争上岗、岗位轮换、考试录用等制度，加快形成公平竞争、充满活力的使用、管理和奖惩机制，激励公务员爱岗敬业、乐于奉献。

　　改进作风，建设勤政廉洁的政府。要弘扬求真务实精神，树立科学的发展观和正确的政绩观，坚持一切从实际出发，按客观规律办事，使各项工作都能经得起实践、群众和历史的检验。坚持办实事，求实效，珍惜民资民力，反对奢侈浪费，勤俭办一切事业。要坚持察实情、讲真话，经常深入基层调查研究，及时发现并解决问题。要切实精简会议、减少文件，狠抓工作落实，促进发展。要强化抓落实的激励和监督机制，健全和完善领导干部抓落实责任制、督促检查制度、定期报告制度、公开通报制度、考评奖惩和责任追究制度，使工作落实制度化、规范化、经常化。要加强廉政建设和反腐败斗争，认真落实廉政建设责任制，进一步完善监督、约束、防范机制。要抓好领导干部任期经济责任审计，加强对政府公共工程、重大社会公益性资金(基金)的审计。加大对违法违纪案件的查处力度，坚决纠正损害群众利益的不正之风，从源头上防治腐败。

　　各位代表，回顾过去，我们对取得的成绩充满自豪；展望未来，我们对贺州的发展前景信心百倍。让我们紧密地团结在以胡锦涛同志为总书记的党中央周围，高举邓小平理论和“三个代表”重要思想伟大旗帜，在市委的领导下，解放思想，抓住机遇，开拓创新，奋力拼搏，扎实苦干，为全面完成今年经济和社会发展的各项任务，为建设繁荣、富裕、文明、美丽的新贺州而努力奋斗！
